# Defining the Role of GLI/Hedgehog Signaling in Chemoresistance: Implications in Therapeutic Approaches

**DOI:** 10.3390/cancers13194746

**Published:** 2021-09-23

**Authors:** Jian Yi Chai, Vaisnevee Sugumar, Ahmed F. Alshanon, Won Fen Wong, Shin Yee Fung, Chung Yeng Looi

**Affiliations:** 1 School of Biosciences, Faculty of Health & Medical Sciences, Taylor’s University, Subang Jaya 47500, Malaysia; jianyi.chai@sd.taylors.edu.my; 2 School of Medicine, Faculty of Health & Medical Sciences, Taylor’s University, Subang Jaya 47500, Malaysia; vaisneveesugumar@sd.taylors.edu.my; 3 Center of Biotechnology Researches, University of Al-Nahrain, Baghdad 10072, Iraq; ahmed.neama@brc.nahrainuniv.edu.iq; 4 Department of Medical Microbiology, Faculty of Medicine, University of Malaya, Kuala Lumpur 50603, Malaysia; wonfen@um.edu.my; 5 Department of Molecular Medicine, Faculty of Medicine, University of Malaya, Kuala Lumpur 50603, Malaysia

**Keywords:** hedgehog pathway, cancer, chemoresistance, hedgehog inhibitors, molecular mechanism

## Abstract

**Simple Summary:**

Chemotherapy is the mainstay of cancer treatment; however, its use is limited due to the emergence of chemotherapeutic drug resistance in cancers. An essential developmental pathway, namely Hedgehog (Hh), has recently emerged as a major modulator of cancer progression and, more importantly, chemoresistance. Preclinical results have implicated Hh signaling as a promising therapeutic strategy for the chemosensitization of Hh-causing cancers. Despite this, most Hh-targeting inhibitors still remain in preclinical stages, and their use in conjunction with chemotherapy in clinical settings is still underdeveloped. To solve this, a better understanding and identification of the relevant mechanisms regulated by Hh signaling to promote chemoresistance in different cancers are necessary to develop more effective treatment strategies. In this review, we outlined the mechanisms by which Hh induces chemoresistance in cancer. Additionally, current therapeutic strategies utilizing a combination of both Hh inhibitors and chemotherapeutics for overcoming chemoresistance in preclinical and clinical settings are also presented.

**Abstract:**

Insight into cancer signaling pathways is vital in the development of new cancer treatments to improve treatment efficacy. A relatively new but essential developmental signaling pathway, namely Hedgehog (Hh), has recently emerged as a major mediator of cancer progression and chemoresistance. The evolutionary conserved Hh signaling pathway requires an in-depth understanding of the paradigm of Hh signaling transduction, which is fundamental to provide the necessary means for the design of novel tools for treating cancer related to aberrant Hh signaling. This review will focus substantially on the canonical Hh signaling and the treatment strategies employed in different studies, with special emphasis on the molecular mechanisms and combination treatment in regard to Hh inhibitors and chemotherapeutics. We discuss our views based on Hh signaling’s role in regulating DNA repair machinery, autophagy, tumor microenvironment, drug inactivation, transporters, epithelial-to-mesenchymal transition, and cancer stem cells to promote chemoresistance. The understanding of this Achilles’ Heel in cancer may improve the therapeutic outcome for cancer therapy.

## 1. Introduction

Cancer is the second leading cause of death and a global burden to public health worldwide. According to the International Agency for Research on Cancer (IARC), in 2018, there were 18.1 million new cases and 9.5 million deaths recorded. Despite the advancement in the cancer treatment approach, cancer incidences remain on the gradual uprise, and it is predicted that by 2040, the incidences of cancer-related cases and deaths would increase to 29.5 million and 16.4 million, respectively [1]. To date, major intervention options for cancer still include surgery, chemotherapy, radiotherapy, targeted therapy, endocrine therapy, and immunotherapy. Among these strategies, chemotherapy remains the mainstay of cancer treatment due to its ability to target fast-growing metastatic cancer cells systematically. Its use in cancer management has a wide variety of purposes; for instance, in adjuvant chemotherapy, drugs such as cisplatin, leucovorin, and oxaliplatin may be administered after surgery or radiotherapy in various cancers to prevent tumor recurrence, for example, in colon cancer [2]. On the other hand, in neoadjuvant chemotherapy, drugs such as anthracyclines are given to patients with advanced breast cancer to reduce the tumor size prior to tumor resection, which may improve clinical response [3]. In some cases, successful chemotherapeutic outcomes can be observed with the remission or the reduction in the size of cancer cells; however, despite chemotherapy being the backbone of cancer treatment, chemoresistance is still responsible for most cancer relapses and metastasis [4,5], representing a major obstacle in cancer therapy. Additionally, long chemotherapy treatment courses are accompanied by unpleasant and adverse side effects that negatively affect one’s quality of life. In light of this, novel anti-cancer therapies targeting molecular events that allow selective drug accumulation in tumor cells over off-target normal cells have been developed, which poses less adverse effect and has significantly improved therapy response for a small subset of patients with identifiable biomarkers [6,7]. More importantly, targeted therapies have been used in combination with chemotherapy to help significantly improve clinical response [8].

A major cause of chemoresistance results from dysregulated evolutionary conserved signaling cascades, such as Wingless (WNT) [9,10], epidermal growth factor receptor (EGFR) [11,12], phosphoinositide-3-kinase (PI3K)/AKT [13,14], extracellular signal-regulated kinase (ERK) [15,16], and nuclear factor-κB (NF-κB) [17,18] pathway, all of which are also known inducers of oncogenesis. As their activation regulates various intrinsic drug resistance mechanisms to promote chemoresistance, researchers have begun focusing their attention on cancer signaling pathways to develop new cancer treatments to improve treatment efficacy. Notably, a new but essential developmental signaling pathway, namely Hedgehog (Hh), has recently emerged as a major mediator of cancer progression and chemoresistance. Despite Hh being a relatively new player in cancer biology, accumulating evidence has strongly implicated Hh and its regulatory mechanism in various forms of oncogenesis in many known cancers [19,20], especially in basal cell carcinoma (BCC) [21,22,23] and medulloblastoma (MB) [24,25], making them an exciting target for cancer treatment. Consequently, ongoing efforts have been made to develop potential therapeutic agents that specifically target the Hh pathway for cancer treatment and research. A representation of such breakthroughs is the development of vismodegib and erismodegib, the U.S. Food and Drug Administration (FDA)-approved Hh inhibitors targeting Smoothened (SMO) that have significantly improved the management of advanced BCC patients with high surgical morbidity and inoperable tumors [26,27]. Additionally, these inhibitors have shown promising results in overcoming various cancer growth in preclinical and clinical trial studies [27,28,29,30,31]. Besides inhibiting tumor growth, preclinical studies have also strongly suggested a promising role of targeting Hh signaling in overcoming chemoresistance in various cancers, making the subject worth exploring. For instance, pharmacological inhibition of Hh signaling with cyclopamine and vitamin D3 sensitized acute myeloid leukemia (AML) cells to vincristine compared to treatment without [32]. Despite these developments, the evidence concerning the use of Hh inhibitors for overcoming cancer chemoresistance in clinical settings is still insufficient—and more importantly, there is inadequate understanding of the molecular mechanisms by which Hh mediates chemoresistance. With that being said, an in-depth understanding of these molecular mechanisms will provide insights on potential therapeutic targets in the hope of developing targeted therapies for mitigating chemoresistance in Hh-causing cancers in the future. Hence, this review will provide an in-depth discussion on the regulatory mechanisms of Hh-mediated chemoresistance that have been reported recently, which includes (1) DNA repair, (2) autophagy, (3) tumor microenvironment (TME), (4) drug inactivation, (5) transporters/drug efflux, (6) epithelial-to-mesenchymal transition (EMT), and cancer stem cells (CSCs). Additionally, this review will also cover the treatment strategies concerning the use of Hh inhibitors with common chemotherapeutics utilized in different studies to improve chemotherapeutic response.

## 2. Hedgehog Signaling

The evolutionary conserved Hedgehog (Hh) signaling pathway, which was initially identified in the fruit fly *Drosophila melanogaster* by Christiane Nüsslein-Volhard and Eric Wieschaus [33], plays a crucial role in embryonic development. The Hh pathway remains dormant in adult tissues but becomes activated when needed, for example, in tissue repair and regeneration [34]. However, its aberrant activation has been linked to cancer development, progression, tumor recurrence, and therapeutic resistance. Thus, understanding the paradigm of Hh signaling transduction is fundamental to provide the necessary means needed to design novel tools for treating cancer related to aberrant Hh signaling.

In humans, Hh signaling is initiated by three Hh ligand homologs, namely Sonic hedgehog (Shh), Indian Hh (Ihh), and Desert Hedgehog (Dhh). Shh is the best-studied ligand among the three proteins due to its major role in development and oncogenesis [35], and it has been detected in a wide range of tissues, including the gut [36]. Hh ligands are synthesized as precursor proteins that undergo autocatalytic cleavage to yield an active NH2-terminal signaling unit with dual lipid modification [37,38]. This modification involves cholesterol modification of the Hh protein at the carboxy terminus and palmitoylation at the amino terminus to ensure proper Hh active signaling and promote its integration into the cell membrane to restrict ligand mobility and regulate the Hh signaling range [39]. The release of lipidated Hh ligands from cell surfaces is facilitated by the combined action of Dispatched (DISP), a 12-pass transmembrane protein with a sterol sensing domain, and Scube2, a secreted glycoprotein [40]. After secretion, Hh ligands form multimers by the association of lipid moieties to allow their interaction with cell surface protein, such as LRP2 and proteoglycans that facilitate their cellular trafficking and internalization into cells and transport to distant sites [41,42,43]. The Hh signaling is initiated upon binding of Hh ligand to the 12-pass transmembrane protein Patched (PTCH1 and PTCH2) receptor [44] to form the Hh-PTCH complex, which is subsequently internalized and degraded in the lysosomes [45]. The binding action alleviates the repression of SMO, a G-protein coupled receptor (GPCR)-like 7-pass transmembrane protein, and with the help of beta-arrestins and kinesin motor protein Kif3A [46,47], promotes its translocation to the primary cilium, a cellular projection from the surface of most cells where the majority of Hh signaling takes place [48]. Additionally, SMO is phosphorylated by several kinases such as protein kinase A (PKA), casein kinase 1 (CK1), and G-protein-coupled receptor kinase 2 (GRK2), which blocks its endocytosis and subsequent degradation as well as stabilizes its active conformation to promote its ciliary accumulation and activation [49,50]. In the absence of Hh ligands, however, PTCH inhibits the translocation of SMO to the primary cilium to repress SMO activity and promote its ubiquitination and degradation via the SUMO-specific peptidases (SENP) [51].

SUFU, a major Hh pathway negative regulator, functions by sequestering glioma-associated oncogene homolog (GLI) in the cytoplasm in the absence of Hh ligands [52]. Sequestered GLI proteins are phosphorylated by PKA, glycogen synthase kinase-3 beta (GSK3β), and CK1 and subsequently recognized by β-transducin repeat-containing protein (β-TrCP), resulting in their ubiquitination and proteasome-dependent proteolytic cleavage to form transcriptional GLI repressors (GLIR) [53,54]. Conversely, SMO activation promotes the translocation of the GLI-SUFU complex from the cytoplasm to the cilia, a necessary step that precedes the dissociation of GLI proteins from SUFU [55]. The dissociation of GLI from SUFU prevents their processing into repressors, favoring the release of full-length GLI or GLI activator (GLIA) [56]. Consequently, GLIA translocates into the nucleus to transcribe target genes such as *CCND1/D2*, *CCNE1*, *MYC*, *BCL2*, *SNAIL*, *NANOG*, and *SOX2*, as well as positive and negative regulators of Hh signaling *GLI1* and *PTCH1* [57,58]. GLI proteins exist as three different homologs, namely GLI1, GLI2, and GLI3. GLI1 lacks the repressor domain and hence acts solely as a transcriptional activator (GLI1A). On the other hand, GLI2 and GLI3 possess both the activator and repressor domain and may function as both activators (GLI2/3A) and repressors (GLI2/3R) determined by their post-transcriptional and -translational processing [59].

While canonical Hh signaling transduces via the typical Hh/PTCH/SMO/GLI route as described above, noncanonical regulation of GLI in GLI-mediated transcriptional regulation is external to the Hh pathway. In support of this, various signaling pathways such as transforming growth factor-beta (TGF-β) [60,61], epidermal growth factor receptor (EGFR) [62], mitogen-activated protein kinases (MAPK) [63], and WNT/β-catenin [64,65] have been reported to induce the expression of GLI proteins independent of SMO. Since both canonical and noncanonical axis culminates in GLI-mediated transcription, GLI protein represents the most promising target for cancer therapy; for instance, drugs that target GLI function such as GANT61 have been shown to exert superior anti-cancer therapy compared to upstream targets [66,67,68]. Anyhow, the expression of both upstream and downstream Hh components, including Shh/Ihh, PTCH1, SMO, SUFU, and especially GLIs, has all been implicated in cancer chemoresistance one way or another, which suggests the availability of one or more potential therapeutic Hh targets for overcoming chemoresistance in Hh-promoting cancers. As the role of noncanonical regulation of GLI in promoting chemoresistance has not yet been sufficiently elucidated, this review will focus majorly on the canonical Hh signaling and the treatment strategies employed in different studies, with special emphasis on the molecular mechanisms and combination treatment in regard to Hh inhibitors and chemotherapeutics.

## 3. Mechanism of Chemoresistance

### 3.1. DNA Repair

The DNA repair machinery is a biological phenomenon by which cells identify and repair DNA damages induced by environmental insults, such as ultraviolet radiation, reactive oxygen species (ROS), mutagens, x-rays, and chemotherapeutic drugs. However, when the DNA repair machinery fails to repair DNA damages such as O6-methylguanine, base N-alkylations, bulky DNA adducts, DNA crosslinks, and DNA double-strand breaks (DSBs) in cells, they undergo imminent cell death due to the activation of the apoptosis pathway [69]. In light of this, novel therapeutic drugs such as alkylating agents temozolomide (TMZ), anti-metabolite 5-Fluorouracil (5-FU), and anthracyclines pemetrexed that induces DNA damages in fast-growing cancer cells have been developed. To overcome this, cancer cells have been known to employ or overexpress DNA repair machinery enzymes to repair DNA damage induced by these chemotherapeutic drugs intensively. For instance, the recruitment of DNA glycosylase, such as SMUG1, excises 5-FU that are incorporated into DNAs and thus abrogates their DNA-induced genotoxicity in cancer cells [70].

Studies from the past have shown that the Hh pathway plays a crucial role in regulating almost all major DNA repair machinery in cancer, including mismatch repair, nucleotide excision repair (NER), direct repair (DR), base excision repair (BER), and DNA double-strand break repair (DSBR) [71]. For instance, Wu et al. reported that SMO inhibition with cyclopamine increased the radiosensitivity (enhanced γH2AX foci) of human pancreatic cancer cells by inhibiting the expression of radiation-induced DNA DSB repair proteins DNA-PKcs and Ku70 [72]. However, despite the abundance of evidence that implicated Hh signaling in the regulation of DNA repair, only a handful of studies have provided a novel link between Hh signaling and its DNA-repair regulatory mechanism in cancer chemoresistance, making it worth emphasizing.

For instance, a novel link between the Hh pathway and the O-6-methylguanine-DNA-methyltransferase (MGMT) repair mechanism in conferring glioma cells TMZ resistance has been reported in several studies [73,74,75]. TMZ is a promising oral alkylating agent that has been used for the treatment of glioblastoma (GBM) for the past few decades due to its ease in administration and its ability to penetrate the blood–brain barrier easily; however, at least half the patients who underwent TMZ treatment developed resistance [76]. This tragic phenomenon is due to the overexpression of MGMT commonly observed in glioma patients, whereby MGMT functions by actively transferring the methyl group from the O6 position of guanine (O-6-methylguanine) induced by TMZ to an active cysteine residue that resides within the protein, thereby leading to TMZ resistance. Past studies have suggested that MGMT may serve as a transcriptional target of GLI1 by virtue of GLI-binding sites within its promoter region [25,77]; however, it was only until recently that evidence has shown that aberrant GLI1 activation regulates MGMT expression to confer chemoresistance in glioma. An earlier study by Li et al. suggested a relation between GLI1 and MGMT expression in promoting GBM cells’ therapeutic resistance, and the inhibition of GLI1 with GANT61 reduced MGMT levels and increased TMZ-induced DNA DSBs [73]. Through bioinformatics and ChiP-qPCR analyses, a later study by Wang et al. revealed that GLI1 acted as a direct transcriptional regulator of MGMT expression in GBM cells by binding to the consensus GLI-binding sequence 5′-GACCACTCG-3′ in the *MGMT* promoter [74]. Additionally, it was revealed that in primary GBM tissues, GLI1 and MGMT levels were markedly elevated and positively correlated. Subsequent analysis revealed that the overexpression of *GLI1* by pcDNA3.1-GLI1 transfection of the GBM A172 cell line expressing low levels of GLI1 resulted in elevated MGMT levels and enhanced TMZ resistance; conversely, the inhibition of Hh/GLI1 signaling with cyclopamine in GBM U251 and U87 cell lines expressing high GLI1 levels led to decreased MGMT levels and enhanced TMZ toxicity. A similar effect was also noted upon the inhibition of Hh-GLI1 in mice GBM xenografts, whereby concurrent treatment with cyclopamine and TMZ significantly inhibited their MGMT expression and tumor burden compared to TMZ alone [74].

In a more recent study, hypoxia was reported to confer TMZ resistance through the hypoxia-inducible factor (HIF-1α)/Shh/GLI1-dependant upregulation of the MGMT DNA repair enzyme. The simulated hypoxic condition of the tumor microenvironment of solid tumors demonstrated that *GLI1* and its downstream target genes (*N*-*myc*, *CCNB1*, and *Bcl*-*2*) were significantly upregulated in several glioma cell lines in a time-dependent manner, and this was positively correlated with MGMT expression. Similar to the study mentioned previously, bioinformatics and ChiP analyses revealed that GLI1 was enriched at *MGMT* promoter regions of GBM cells, and the GLI1-binding consensus sequence was suggested to be 5′-GACCACCCA-3′. Subsequent analyses revealed that HIF-1α, a transcriptional regulator of cellular responses to hypoxia, induced the upregulation and autocrine secretion of Shh, thereby promoting the translocation of GLI1 to the nucleus upon Hh signal transduction. Conversely, the degradation of HIF-1α by oroxylin A or the inhibition of Hh/GLI1 signaling by cyclopamine led to the downregulation of the HIF-1α/Hh pathway and the increased expression of SUFU, thus restoring glioma cells’ chemosensitivity to TMZ [75]. Taken together, hypoxia may potentially regulate TMZ resistance in glioma cells through the HIF-1α/Shh/GLI1 axis. Consequently, GLI1 upregulates MGMT expression by binding to several consensus GLI-binding sites within the *MGMT* promoter to promote TMZ resistance.

To date, the most common treatment of colorectal cancer still involves the use of 5-FU, a synthetic fluorinated pyrimidine that interferes with nucleoside metabolism by incorporating itself into DNA and RNA [78]. In various studies, it has been reported that the Hh pathway and NBS1 of the Mre11/RAD50/NBS1 (MRN) double-strand break repair (DSBR) complex plays an important role in colorectal cancer (CRC) progression and resistance. For instance, elevated levels of NBS1 expression led to worse overall survival and poorer response in rectal cancer patients receiving neoadjuvant therapy [79]. Similarly, high levels of GLI1 were associated with a higher incidence of tumor relapse in CRC patients who underwent 5-FU based chemotherapy [80]. In light of these findings, a novel link between GLI1 and NBS1 in conferring 5-FU drug resistance in CRC was revealed. In CRC patients receiving 5-FU treatment, GLI1 and NBS1 were expressed synchronously, and their higher expression levels were associated with a poorer prognosis overall. Further analyses revealed that elevated GLI1 and NBS1 protein levels led to reduced DNA damage and stronger drug resistance in the 5-FU treated HT29 cell line; conversely, the HCT-116 cell line expressing low GLI1 and NBS1 levels was susceptible to 5-FU-induced DNA toxicity. Indeed, the inhibition of GLI1 by SIR-38832 resulted in a reduction in total NBS1 levels and decreased colocalization of NBS1 and MRE11 that, in turn, impaired MRN complex function and restored chemosensitivity in 5-FU resistant HT29 cell lines and xenografts. Through chromatin-ChiP analysis, it was revealed that *NBS1* was a direct transcriptional target of GLI1, as indicated by the enrichment of GLI1 binding at the *NBS1* promoter containing the constitutive GLI-binding site 5′-GACCACCCA-3′ [81], further enforcing the role of GLI1 in regulating NBS1 function to promote chemotherapeutic drug resistance.

The Hh pathway has also been known to influence cisplatin resistance in ovarian cancer (OC) by regulating the DNA excision repair protein ERCC1, an endonuclease that functions in the nucleotide excision repair (NER) pathway. Initial studies by Li et al. revealed that ERCC1 is vital for the repair of platinum-induced DNA damage after cisplatin exposure in OC cells, leading to cisplatin resistance, and the ERCC1 expression was mediated by the phosphorylation of c-jun, which activates the activator protein 1 (AP1) to increase ERCC1 transcription [82,83]. A further study by the researchers revealed that GLI1 was significantly upregulated in cisplatin-resistant OC A2780-CP70 cell lines compared to cisplatin-sensitive OC A2780 cell lines. Consequently, elevated GLI1 increased the phosphorylation of c-jun at Ser63/73 (pro-growth, anti-apoptotic), which upregulated ERCC1 expression that, in turn, reduced platinum-induced DNA adducts and enhanced cisplatin resistance. Conversely, treating the resistant cells with anti-GLI1 shRNA shifted the c-jun Ser 63/73 phosphorylation dominant pattern to a Thr91/93 (pro-apoptotic) phosphorylation dominant pattern, which reduced ERCC1 expression and consequently restored cisplatin sensitivity, as shown by enhanced supra additive cell killing and increased platinum-DNA adduct levels. Additionally, treatment with anti-GLI shRNA reduced Shh protein expression by five-fold in the cisplatin-resistant sublines, suggesting an autocrine feedback loop where GLI1 contributes to increased Shh production ligand and consequently Hh pathway activation to promote chemoresistance [84].

**Figure 1:**
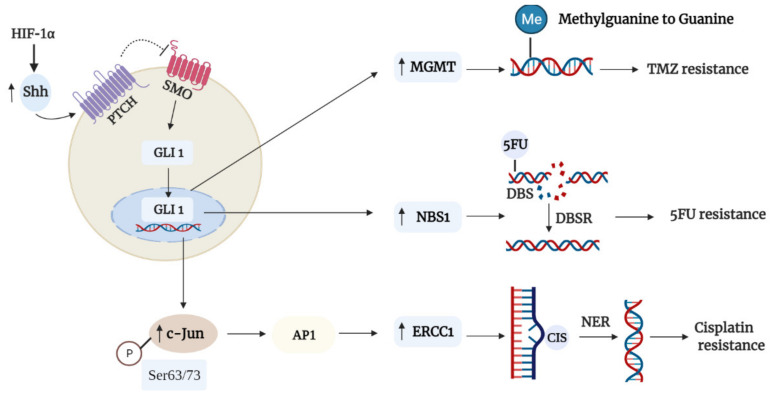
A schematic representation of the Hedgehog (Hh) signaling in the regulation of different DNA repair mechanisms to promote chemoresistance. Under the hypoxic condition, HIF-1α stimulation triggers the autocrine secretion of Shh, which activates canonical Hedgehog-glioma-associated oncogene homolog 1 (Hh-GLI1) signaling in tumor cells to induce high levels of MGMT. Consequently, MGMT repairs O-6-methylguanine lesions induced by TMZ, which results in TMZ resistance. Activation of Hh-GLI1 signaling also upregulates NBS1 of the MRN complex as well as promotes c-Jun phosphorylation (Ser63/73) and AP1-mediated upregulation of ERCC1 to repair DNA breaks induced by 5-FU and cisplatin, respectively.

These results demonstrated that canonical Hh-GLI signaling plays a crucial role in regulating DNA repair systems to reduce chemotherapeutic drug-induced genotoxicity. Figure 1 summarizes the DNA repair mechanisms which Hh signaling utilizes to promote chemoresistance described in this section. With that being said, it would be interesting to explore if Hh signaling may also regulate other DNA repair pathways to promote chemoresistance to other DNA-targeting drugs that have not been covered.

### 3.2. Autophagy

Autophagy is a self-degradative cellular process that helps maintain cellular metabolism and survival through the degradation and recycling of cytoplasmic contents in exchange for nutrients and energy. However, a growing body of evidence has also implicated the role of autophagy in tumorigenesis, treatment, and chemoresistance of cancer. It has been well-established that in cancer cells, autophagy is considered to be a double-edged sword, as its activation has been shown to not only promote tumorigenesis by promoting cancer-cell survival and tumor growth but also inhibit tumorigenesis by inducing autophagic cell death. For instance, previous studies have reported that UV radiation resistance-associated gene (UVRAG) interacts with Beclin-1, a protein that is involved in the formation of the phagophore, to function as tumor suppressors by positively regulating autophagy-dependent cell death in various cancers such as colon, gastric, breast, and prostate cancer [85,86,87,88]. Conversely, in apoptosis-defective immortalized baby mouse kidney epithelial (iBMK) cells, the overexpression of Beclin-1 activates the autophagy-dependent survival pathway and consequently promotes the survival of iBMK cells to metabolic stress when apoptosis is inactivated [89]. Similarly, autophagy misactivation has also been shown to either promote or diminish chemoresistance in cancer. For example, autophagy has been shown to promote drug resistance to various chemotherapeutic drugs such as cisplatin, sorafenib, and carboplatin in ovarian, esophageal, and liver cancers [90,91,92]. On the other hand, treatment with chemotherapeutic drugs such as imatinib and TMZ induced senescence and autophagic cell death in blood and brain cancers [93,94].

In recent years, the role and mechanism of the Hh pathway in autophagy have been extensively studied in human diseases and cancer. Evidently, the Hh pathway has been known to either inhibit or promote autophagy in different cancers to promote cell survival. In several cancers such as gastric, ovarian, colon cancer, and glioma, Hh pathway activation suppressed autophagy and the accumulation of SQSTM1/p62 and LC3-II, which enhanced the growth of these cancer cells [95]. Conversely, KRAS induced autophagy via the noncanonical KRAS–PI3K–AKT1–GLI3–VMP1 axis to enhance tumorigenesis and cancer progression in KRAS-driven tumors [96]. Individually, both Hh and autophagy have been extensively implicated in the regulation of chemotherapeutic drug resistance, and Hh signaling has also been shown to play a significant role in autophagy regulation. Thus, it is not surprising that Hh may very well promote chemoresistance through the regulation of autophagy.

In bladder cancer (BC) cells, a novel link between Hh and autophagy-induced chemoresistance was reported by Amantini et al. Evidently, Capsaicin (CPS), a bioactive alkaloid found in chili peppers of the plant genus *Capsicum*, was reported to induce oxidative stress that triggers protective autophagy in BC cells, thus rescuing them from imminent cell death [97]. Moreover, the knockdown of *Beclin-1* using siRNA abrogates CPS-induced autophagy, which significantly reduced BC cell growth by causing necrotic cell death. Later, it was discovered that the Hh gene members (*Dhh* and *PTCH2*) were upregulated in association with autophagy activation (increased LC3II/LC3I ratio) in CPS-treated BC cells, and suppression of the Hh pathway through siPTCH2 resulted in decreased autophagy activation (reduced LC3II/LC3I ratio). Further analysis revealed that the Hh-induced autophagy in CPS-treated BC cells was linked to stronger resistance to standard chemotherapeutic agents (mitomycin C, gemcitabine, and doxorubicin) used in BC therapy. Compared to CPS-untreated cells with low autophagy activity, CPS-treated BC cells were two-three fold more resistant to the chemotherapeutic drugs mitomycin C, gemcitabine, and doxorubicin, as shown by the increase in cell growth in the CPS-treated group [97]. Overall, CPS triggers autophagic survival in a Hh-dependent manner to promote chemotherapeutic drug resistance in BC cells.

However, in GBM, ROS-induced autophagy mediated by Hh pathway inhibition triggered autophagic cell death in response to TMZ treatment. GANT-61 and TMZ combined treatment led to significantly increased ROS production, acidic vesicular organelle (AVO), and *Beclin*-*1* expression in several GBM cell lines compared to TMZ treatment alone, implying that Hh inhibition synergizes with TMZ to enhance autophagy activation via ROS signaling in GBM cells. Consequently, the induction of autophagy in GANT-61 treated GBM cells sensitized them to TMZ, as shown by increased cell death, loss of membrane integrity, and DNA fragmentation [98]. In contrast to the study mentioned previously, Hh does not activate autophagy to promote cancer cell survival, but rather it plays an inhibitory role in autophagy activation to prevent autophagy-dependent cell death in response to chemotherapeutic treatment.

### 3.3. Tumor Microenvironment

In a solid tumor bulk, it consists not only of a heterogeneous pool of cancer cells but also includes a variety of cellular and non-cellular components such as fibroblasts, immune cells, soluble growth factors, extracellular matrix (ECM), and the vasculature system, which is collectively known as the tumor microenvironment (TME) [99]. Depending on how tumor cells interact with their microenvironment, it may profoundly impact tumor initiation, progression, and ultimately metastasis, or it may lead to tumor eradication. For instance, hypoxic conditions were shown to accumulate HIF-1α, which at sufficient levels activates the Hh pathway in pancreatic cancer cells to promote EMT and consequently invasion [100]. In the context of pH modification, the acidic microenvironment resulting from tumor-derived lactic acid was shown to prevent lactic acid export from cytotoxic T-cell lymphocytes (CTLs), impairing their metabolism and consequently immune function such as proliferation, cytokine production, and infiltration [101]. In turn, loss of T-cell function results in decreased immune clearance of tumor or aberrant cells [102]. Initially, it was thought that tumor cells’ ability to resist chemotherapeutic agents was only due to aberrant cellular responses within the tumor cell itself. However, now it is clear that TME plays a major role in shaping the therapeutic responses and chemoresistance of tumors, which explains why most cancer cells’ responses to chemotherapeutic agents in vitro fail to correlate with the same tumor-derived cells in vivo [103].

Commonly, TME-induced chemoresistance involves the paracrine crosstalk between stromal and cancer cells through various oncogenic pathways, which can induce gene expression changes that lead to the reshaping of tumor milieu to foster tumor progression and confer stronger resistance to cancer therapeutics. One of the common consequences of such reciprocal crosstalk between stromal and epithelial cells involves Hh signaling, a new major player in TME remodeling, whereby the secretion of Hh ligands from a subset of epithelial cancer modulates the stromal cells to reshape the ECM in a way that promotes a more drug resistant phenotype in cancer cells. For instance, an earlier study by Hui et al. found that Hh ligands secreted from triple-negative breast cancer (TNBC) epithelial cells activate Hh signaling in stromal cancer-associated fibroblasts (CAFs) to promote therapeutic resistance [104]. In light of this, a further study by the same group found that Hh ligand secreted from M6-Hh (Hh expressing murine TNBC) cells was found to reprogram CAFs to provide a supportive niche for the acquisition of a more chemoresistant, CSC phenotype in surrounding TNBC cells via extensive remodeling of the ECM. Evidently, the Hh pathway induced CAFs to increase the production of ECM protein, such as collagen (Col2a1, Col3a1, Col4a1) and metalloproteinases (Mmp3, Mmp13, Mmp15), which remodels the ECM via increased fibrillar collagen deposition and crosslinking at the tumor-stromal interface. The ECM remodeling led to increased collagen mechano- and focal adhesion kinase (FAK) signaling in M6 cells, which conferred a more chemoresistant CSC-like phenotype, as shown by increased clonogenic capacity and stem cell markers (Id3, Itgb3, Krt6) [105,106].

Additionally, mechanistic studies also revealed Hh-mediated upregulation of fibroblast growth factor 5 (FGF-5) as an additional mechanism contributing to CSC plasticity. In support of this, RNA-seq analysis revealed *FGF*-*5* to be strongly upregulated in Hh-activated CAFs, which caused increased phospho-FGFR activation in adjacent M6-Hh epithelial cells. Subsequently, this led to an increase in the CSC phenotype of these cells, as shown by the robust upregulation in stem cell markers (Id3, Sox10) and sphere-forming capacity. Consequently, increased fibrillar collagen deposition and FGF-5 expression combined, resulting from CAF activation by M6-Hh cells secreting Hh ligands, led to the formation of a supportive CSC niche that was more chemoresistant. Indeed, stromal Hh pathway inhibition with GDC-0449/NVP-LDE225 reduced FAK activation, FGF-5 expression, and CSC markers in M6-Hh tumors and patient-derived xenografts that, in turn, sensitized them to docetaxel. Moreover, in a phase-I clinical study, combination therapy with sonidegib and docetaxel resulted in an overall improved clinical response in TNBC patients, up to the extent of the complete resolution of lung metastases. However, this was mainly restricted to patients with high stromal and paracrine Hh pathway activation exhibiting high phospho-FGFR and collagen deposition [105,106], further enforcing the role of paracrine Hh signaling between TNBC stromal and tumor cells as a novel mediator of CSC plasticity and chemoresistance. Of note, although both FAK signaling and phosphor-FGFR activation promote CSC phenotype in TNBC, whether their signaling was co-dependent in promoting CSC phenotype and chemoresistance in TNBC has not been elucidated.

Besides crosstalk between cancer and fibroblast or immune cells, the TME also provides many cues that affect the perfusion and vasculature surrounding the solid tumor. This effect is most apparent in pancreatic ductal adenocarcinoma (PDA), where the vasculature is profoundly affected by the degree of desmoplasia. Many PDA tumors are characterized by hypovascularity and hypoperfusion as a result of tumor desmoplasia, which negatively impacts the pharmacodelivery of drugs to tumor tissues [107]. Despite mounting evidence suggesting PDA’s angiogenic independence in tumor growth, the molecular mechanism behind how extensive desmoplasia leads to hypovascularity and a subsequent decrease in chemotherapeutic drug delivery has not been sufficiently elucidated. Numerous studies have pointed toward the Hh pathway’s role in modulating the PDA TME, as paracrine Hh signaling from neoplastic to stromal cells promotes stromal desmoplasia [108,109], and high Hh activity has been detected in stromal cells surrounding Hh-expressing tumor epithelium [110]. Indeed, a KrasG12D/+; LSL-Trp53R172H/+; Pdx-1-Cre (now referred to as KPC) mice model of PDA treated with SMO inhibitor, IPI-926, demonstrated enhanced desmoplastic stroma depletion, characterized by densely packed ductal tumor cells, as well as decreased collagen I and α-smooth muscle actin (αSMA) positive stromal myofibroblasts proliferation, and this effect was even more profound than gemcitabine treatment. Notably, KPC mice were not responsive to the treatment of gemcitabine alone; however, SMO inhibition with IPI-926 resulted in a marked increase in microvessel density (MVD) and improved perfusion that, in turn, correlated with more effective delivery of gemcitabine to KPC mice tumor tissues, as indicated by elevated intratumoral levels of gemcitabine metabolites dFdCTP. As a result, KPC mice that were sensitized to gemcitabine showed an overall improved survival rate and transient tumor stabilization, as well as decreased metastasis [111].

**Figure 2:**
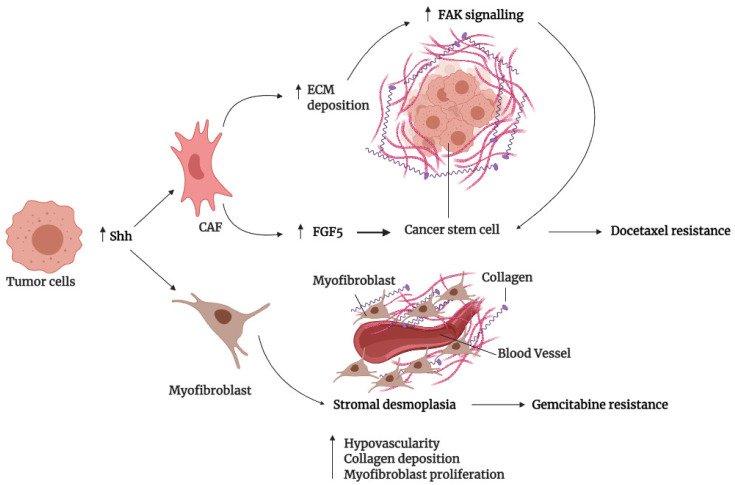
A simplified illustration of the Hh signaling in the reprogramming of TME to confer chemotherapeutic drug resistance. Paracrine Hh signaling between tumor and stromal cells (CAF and myofibroblast) via tumor-derived shh induces stromal cells to reshape TME via increased ECM deposition (e.g., collagen), which leads to increased CSC niche and stromal desmoplasia (also characterized by increased myofibroblast and decreased blood vessel). Additionally, Hh-activated CAF also secretes FGF5, which binds to FGFR on adjacent tumor cells to induce CSC phenotype. Consequently, the increase in CSC niche and decreased blood vessel formation resulting from stromal desmoplasia enhance innate resistance to cancer therapeutics and decrease drug delivery, resulting in decreased chemotherapeutic drug response.

Overall, Hh signaling, through the extensive remodeling of the ECM, can confer chemoresistance to tumor cells through modulation of the CSC niche and drug delivery. Of note, Hh signaling modulation of chemoresistance is not restricted to autocrine signaling alone, as the inhibition of paracrine signaling between Hh ligand-producing tumor cells and surrounding stromal cells has proven effective in improving TME to restore chemotherapeutic drug sensitivity. Figure 2 depicts the role of paracrine Hh signaling in TME modulation for conferring drug resistance described in this section.

### 3.4. Drug Inactivation

Drug inactivation or modification is one of the strategies which cancer cells employ to resist chemotherapeutic agents. One of the common mechanisms involves the phase II glucuronidation reaction, which is the most crucial detoxification pathway for a broad spectrum of drugs. The glucuronidation pathway consists of several types of UDP-glucuronosyltransferases (UGTs) that function by transferring glucuronic acid from the cofactor uridine-5′-diphospho-α-D-glucuronic acid (UDPGA) to a substrate, rendering it more water-soluble and readily eliminated from the body [112]. When this pathway is overexpressed in cancer cells, it results in a more efficient elimination of chemotherapeutic drugs from the body, rendering chemotherapy ineffective.

UGTs have been shown to mediate oncogenic pathways to promote cancer progression [113], but the notion that oncogenic pathways may regulate UGTs remains to be explored. To date, the Hh pathway remains one of the only few oncogenic pathways that have been properly elucidated in terms of its role in UGTs regulation. For instance, GLI1 protein levels were highly elevated in AML relapse patients after ribavirin monotherapy or ribavirin and low-dose cytarabine (Ara-C) combination treatment, suggesting elevated GLI1 was associated with acquired resistance [114]. Ribavirin acts by inhibiting the function of eukaryotic translation initiation factor 4E (eIF4E) [115,116]; conversely, GLI1 overexpression reduced the levels of ribavirin that, in turn, decreased the formation of eIF4E–ribavirin complexes, thereby conferring chemoresistance. Mechanistically, it was found that GLI1 mediated the UGT1-A dependent glucuronidation of ribavirin and Ara-C in acquired resistant cell lines, which enhanced ribavirin and Ara-C glucuronides formation and subsequent elimination from the cells. Indeed, GLI1 pharmacological and genetic inhibition with GDC-0449 and siGLI1, respectively, overcomes drug resistance by reducing UGT1-A protein levels and consequently UGT1-A dependent glucuronidation of ribavirin and Ara-C, thus restoring their intracellular drug activity. Similarly, in primary AML specimens that failed induction chemotherapy, inhibition of the Hh-GLI1 axis with GDC-0449 potentiated the effects of ribavirin and Ara-C but not in primary specimens from healthy volunteers, suggesting a major role of Hh signaling in conferring drug resistance through inducible glucuronidation [114].

In a later study, GLI1-mediated glucuronidation was further shown to elicit a broad-spectrum multidrug resistance profile in acquired resistant cell lines, as its upregulation conferred drug resistance to approximately 40 compounds, which includes FDA-approved drugs such as methotrexate, venetoclax, 5-FU, sunitinib, and idarubicin, all of which have shown to be substrates of UGT1A enzymes. Similarly, the inhibition of GLI1 restored the drug sensitivity to the drugs above, which was associated with the downregulation of UGT1A protein levels. Of note, GLI1 does not affect *UGT1A* transcript levels but rather its protein stability via the chaperone calreticulin. Indeed, calreticulin was consistently upregulated in GLI1-overexpressing resistant cells, and calreticulin inhibition reduced UGT1A protein levels and subsequently restored drug sensitivity [117].

**Figure 3:**
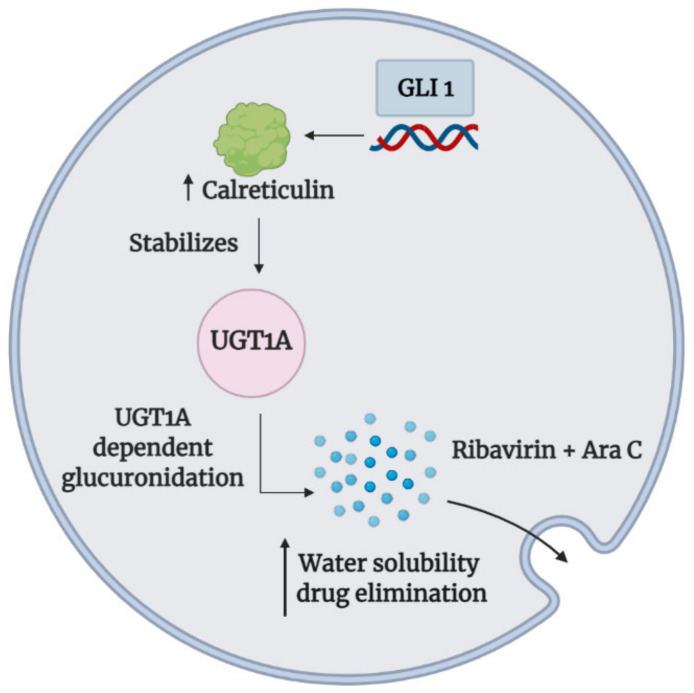
A simplified illustration of GLI1 in the regulation of UGT1A-dependent glucuronidation of ribavirin and cytarabine (Ara-C, cytosine arabinoside). GLI1 upregulates the chaperone calreticulin, which stabilizes UGT1A protein levels to promote UGT1A-dependent glucuronidation of ribavirin and Ara-C. This results in the formation of glucuronides that are more water-soluble and, therefore, more readily excreted.

These results revealed a strong role of GLI1 in promoting chemotherapeutic drug resistance via glucuronidation-dependent drug inactivation. However, there is still much that needs to be achieved to establish a further Hh signaling role in drug inactivation—not just in glucuronidation but also other aspects of drug modification. These observations open up avenues in determining what other cancer therapeutics may be modified by Hh signaling, and if so, via what classes of drug inactivation mechanisms. Figure 3 summarizes the glucuronidation mechanism which GLI1 utilizes to inactivate chemotherapeutics described in this section.

### 3.5. Transporters

The overexpression of ATP-binding cassette (ABC) transporters is primarily responsible for the acquisition of multi-drug-resistance (MDR) phenotype in cancer cells, a phenomenon whereby enhanced efflux of drugs leads to increased resistance towards diverse cancer therapeutics. The ABC transporters are classified into seven subfamilies, namely ABCA-ABCG [118]. Among them, P-glycoprotein (P-gp/MDR1/ABCB1), MDR-associated protein 1(MRP1/ABCC1), and breast cancer resistance protein (BCRP/ABCG2) are better defined in terms of their roles in regulating chemoresistance [119]. Additionally, these ABC transporters are regulated by several oncogenic pathways such as NF-κB, TGF-β, PI3K/AKT, EGF [120], and Hh [121] to promote drug efflux and consequently chemoresistance in cancer. Among these oncogenic pathways, Hh signaling is one of the better well-characterized pathways in terms of its role in regulating ABC transporters across various cancers, which adds to its utmost importance in promoting chemoresistance.

An earlier study by Steg et al. showed that the upregulation of ABCB1 levels by aberrant Hh signaling decreased the sensitivity of OC cells to paclitaxel, an effect that can be reversed upon SMO inhibition with LDE225 [122]. However, the exact Hh element responsible for the ABCB1-mediated chemoresistance in OC was not elucidated until much later, whereby a study by Zhang et al. revealed GLI2 to be the main culprit involved in OC chemoresistance [123]. In support of this, Shh, GLI2, and ABCB1 were found to be significantly elevated in the cisplatin-resistant OC SK-OV-3 cell line, and selective *GLI2* knockdown or GLI2 inhibition with GANT-61 decreased ABCB1 levels, which was accompanied by elevated cisplatin-induced DNA damage, evident by increased levels of DNA damage marker, H2AX. N-Shh conditioned media treatment of ES-2 and SK-OV-3 cells led to a time-dependent increase in ABCB1 protein levels. Furthermore, transfecting *GLI2* overexpression vector into SK-OV-3 cells restored both the transcript and protein levels of ABCB1, an effect which was not seen in a parallel GLI1 overexpression model, suggesting that Hh-mediated ABCB1 upregulation was exclusive only to GLI2. To further enforce the role by which GLI2 promotes cisplatin resistance via ABCB1, the overexpression of GLI2 in the OC ES-2 cell line reduced their sensitivity to cisplatin, and this effect was abrogated upon ABCB1 inhibition with verapamil, a potent ABCB1 inhibitor [123]. Overall, the results suggest that Shh-GLI2 is a strong inducer of ABCB1 expression to promote chemotherapy resistance in OC.

However, in a study by Chen et al., *ABCB1* was found to be a direct transcriptional target of GLII. Transfecting the ABCB1 promoter segment using the pGL4.32 luciferase construct in endogenous GLI1-overexpressing A2780 and OVCAR3 cell lines led to significantly higher luciferase activity [124]. Additionally, transfecting the DNA probe containing the GLI1 consensus binding sequence of the *ABCB1* promoter into the endogenous GLI1-overexpressing A2780 cell line led to shifted bands (EMSA) in nuclear extracts. Interestingly, these OC cells had low levels of ABCB1; but when induced into spheroids, GLI1 and ABCB1 levels were significantly elevated, which conferred resistance to paclitaxel and cisplatin [124]. Of note, CSCs/spheroids are known to employ drug efflux pumps, including ABCB1, to promote drug efflux and CSC maintenance [120]. The conflicting findings between the two studies above may have been attributed to the different cell lines used. Zhang et al. focus specifically on the cisplatin-resistant SK-OV-3 [123]; whereas Chen et al. focus specifically on the A2780 and OVCAR3 [124]. Additionally, cisplatin-resistant SK-OV-3 was found to overexpress GLI2 and ABCB1; whereas A2780 and OVCAR3 were found to overexpress GLI1 and ABCG2 instead. Nevertheless, these studies suggest that in OC, the activation of Hh signaling represents a general regulatory mechanism by which cancer cells enhance drug efflux to promote chemoresistance.

Besides OC, Cui et al. reported that Shh overexpression or siRNA-mediated knockdown of *Shh* led to increased and decreased PTCH1 expression and cisplatin-induced cytotoxicity of esophageal squamous cell carcinoma (ESCC) KYSE510 cells, respectively [125]. Furthermore, continuous cisplatin treatment increased Shh signaling as shown by increased GLI1 nuclear translocation and PTCH1 levels, which in turn upregulate the expression of ABCB1 to reduce cisplatin enrichment in the ESCC ALDH+ TE-1 cell line. Conversely, Hh inhibition with dihydroartemisinin (DHA; a classical antimalarial drug) completely abrogated these effects and restored cisplatin accumulation in TE-1 cells [125]. In the myeloid leukemia Lucena-1 cell line, Hh signaling upregulates ABCB1 expression to promote vincristine resistance, and Hh pathway inhibition with cyclopamine, Vitamin D3, or GANT-61 restored sensitivity to several chemotherapeutics [32]. Lastly, Das et al. reported that osteopontin (OPN), a bone matrix protein, non-canonically activates GLI1 via GSK3β inhibition to promote GLI1 nuclear translocation and GLI1-dependent upregulation of ABC transporters (ABCB1 and ABCG2) and consequently drug resistance to doxorubicin, paclitaxel, and cisplatin by reducing intracellular drug retention in breast cancer cell lines [126]. In support of this, complete inhibition of GSK3β with MeBIO caused a significant increase in GLI1 expression, whereas the introduction of constitutively active GSK3β by the transfection of breast cancer cells enhanced drug retention ability that is abrogated by OPN treatment. Conversely, the silencing of GLI1 and OPN enhanced the sensitivity of breast cancer cells to all three chemotherapeutics. Interestingly, treatment with cyclopamine significantly decreased ABCB1 and ABCG2; but when treated together with OPNi (silencing), it resulted in greater suppression of the ABC transporter protein expression, suggesting dual mechanisms of canonical and noncanonical regulation of GLI in promoting chemoresistance [126].

Hh signaling has also been shown to regulate ABCG2 expression to confer 5-FU and cisplatin resistance in gastric cancer (GC). The RT-qPCR analysis revealed that the treatment of GC cells with 5-FU and cisplatin markedly elevated the levels of *GLI1* and *GLI2*, which was accompanied by increased ABCG2 expression. Additionally, GC patients with high *GLI1*/*GLI2* and *ABCG2* signatures were associated with a shorter overall survival (OS) and higher cancer relapse, suggesting that higher expressions of these genes predicted a poorer outcome in GC patients who underwent 5-FU and cisplatin-based chemotherapy. Mechanistically, ChiP analysis revealed that GLI1/GLI2 regulates *ABCG2* at the promoter level by binding to GLI-binding consensus site GACCACCCA. Indeed, *GLI1*/*GLI2* knockdown significantly downregulated the expression of ABCG2, which restored the sensitivity of GC cells to both 5-FU and cisplatin [127]. In support of this finding, Yu et al. also reported that through ChiP analysis, GLI1 directly regulates the expression of *ABCG2* by binding to the same GLI-binding consensus site within the promoter to maintain putative gastric CSCs and to mediate cisplatin resistance in GC cell lines and xenografts [128]. Notably, a study by Yao et al. demonstrated a noncanonical regulation of GLI1 by p-AKT/mTOR signaling to promote cisplatin resistance in GC cell lines which can be reversed by the inhibition of GLI1 or p-AKT activity [129], and together with the previous data, suggests that cisplatin resistance can be regulated by noncanonical regulation of GLI function. In a separate study, Xu et al. found that GLI1 promoted doxorubicin drug resistance in CD44+/Musashi+ gastric CSCs and mice xenografts by mediating the ABCG2-dependent efflux of the chemotherapeutic, and *GLI1* knockdown or inhibition of GLI1 function with GANT-61 reversed the above effect [130].

Besides GC, an earlier study by Chen et al. also reported *ABCG2* as a direct transcriptional target of GLI1 in OC via the same GLI-binding consensus site as mentioned above, and its upregulation conferred resistance to both cisplatin and paclitaxel [124]. In pancreatic cancer (PANC-1) tumorspheres exhibiting stemness characteristics, the inhibition of Hh signaling with cyclopamine decreased ABCG2 expression and reversed gemcitabine resistance [131].

GLI2 of the Hh signaling pathway was reported by Ding et al. to regulate the ABCC1 transporter in Huh-7 DN (CD133-/EpCAM-) and trans (CD133−/EpCAM− transwell-selected; EMT phenotype) hepatoma subpopulations to confer sorafenib drug resistance [132]. Previously, the researchers demonstrated that the Huh-7 DN subpopulation with upregulating Hh pathway activity (PTCH1 and GLI2) was associated with enhanced EMT acquisition, increased spheroid formation, and higher resistance to cisplatin, doxorubicin, and sorafenib [133]. Further study revealed that GLI2 and ABCC1 expression (but not ABCB1 and ABCG2) were overexpressed in the Huh-7 DN and trans subpopulations associated with increased sorafenib resistance. The inhibition of Hh signaling at the SMO level with LDE225 or itraconazole inhibits the expression of GLI2 (but not GLI1), which, in turn, downregulated the ABCC1 expression in Huh-7 DN and trans subpopulations. This suggests that GLI2 acts as a primary mediator of sorafenib drug sensitivity in these subpopulations, at least in part, through ABCC1 transporter regulation [132]. Further study by the same group also revealed that GLI1/GLI2 binds to the GLI1-binding consensus sequence within the *TAP1* (gene encoding ABCB2 protein) promoter to initiate its transcription in Huh-7 DN and trans subpopulations, which consequently promotes drug resistance to cisplatin, doxorubicin, and sorafenib. Conversely, the knockdown of *GLI1* or *TAP1* via an RNAi approach or the inhibition of GLI1/GLI2 function with GANT61 markedly improved the subpopulations’ sensitivity to all three cancer therapeutics [134].

Besides ABC transporters, multiple transport proteins such as octamer-binding protein OCT1, OCT2 and OCT3, and copper transporter CTR1 have been known to promote the influx of cisplatin to promote cytotoxicity. On the other hand, ATPase copper transporter ATP7A promotes the efflux of cisplatin to confer cisplatin resistance. A study by Amable et al. has revealed that, according to the EMSA, GLI1 binds to the promoter of all five of these genes responsible for cisplatin transport, and its inhibition can both reduce cisplatin uptake and influx in OC. However, as the inhibition of GLI1 had a more profound effect on drug efflux, it ultimately led to increased cisplatin accumulation in the nucleus and cytotoxicity [135].

**Figure 4:**
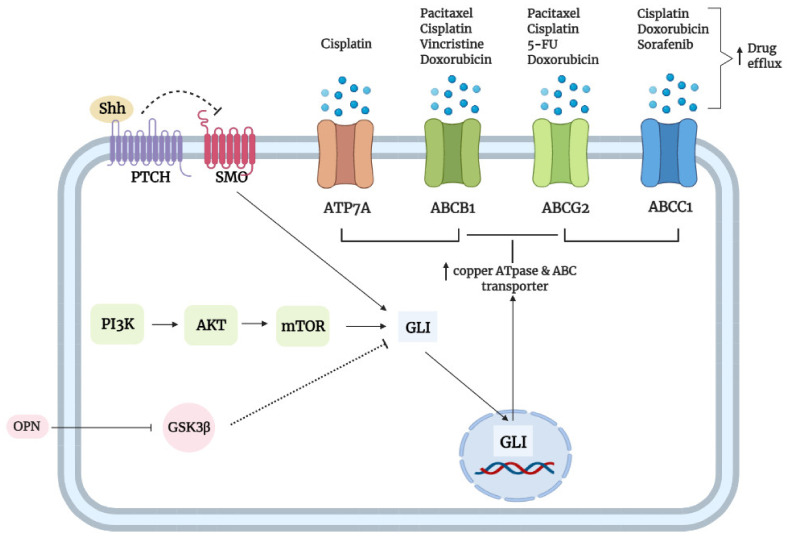
A simplified representation of canonical and noncanonical regulation of GLI in promoting transporter-mediated drug efflux. In the canonical axis, Shh binds to PTCH1, resulting in the alleviation of SMO repression and subsequent GLI activation. In the noncanonical axis, OPN inhibits the negative regulator of GLI, GSK3β, alleviating the repression of GLI1 function. Activated GLI then translocates into the nucleus, inducing the transcriptional upregulation of copper ATPase and ABC transporters to enhance drug efflux and, consequently, reduce intracellular drug levels.

Taken together, these studies substantiate a vital role of Hh signaling in regulating various transporters, possibly at the promoter level, to confer chemotherapeutic drug resistance. Additionally, many of the reported CSCs (as described above) or EMT cells also utilized Hh signaling as a means to upregulate various transporters to promote chemoresistance. Thus, targeting Hh signaling holds promise for the chemosensitization of tumors by maintaining high intracellular drug activity levels. Figure 4 summarizes the drug inactivation and drug efflux mechanisms which canonical Hh-GLI signaling or non-canonically activated GLI utilizes to promote drug elimination described in Section 3.4 and this section, respectively.

### 3.6. Epithelial-to-Mesenchymal Interaction

EMT is an essential physiological process in which epithelial cells lose their epithelial phenotype and acquire mesenchymal characteristics. This transition is characterized by the reversible loss of cell polarity and cadherin-mediated cell adhesion, as well as enhanced motility and invasiveness [136]. Physiologically, EMT governs many biological phenomena, such as embryogenesis, wound-healing, and stem cell biology, to ensure proper organ development and tissue homeostasis. Similarly, Hh signaling has also been shown to play an indispensable role in mediating these processes through EMT induction by regulating several key EMT-inducing transcription factors, such as TWIST1/2, SNAI1, SLUG, and ZEB1/2 [57]. These transcription factors mediate the epithelial to mesenchymal switch by shutting down the expression of epithelial markers (e.g., E-cadherin) while concomitantly upregulating mesenchymal markers (e.g., vimentin, N-cadherin). However, the induction of EMT as a consequence of aberrant Hh signaling has been implicated in cancer cell metastasis and invasion. Moreover, a growing body of evidence has also implicated EMT induction in the acquisition of chemoresistance to a variety of chemotherapeutics, such as FU [137], cisplatin [138], and vincristine [139]. Of note, EMT does not directly lead to chemoresistance, per se, but its acquisition has been commonly associated with upregulated drug resistance mechanisms. For instance, the upregulation of EMT transcription factors such as TWIST1, SNAI1, and ZEB1 has been shown to regulate the expression of several ABC transporters to promote chemoresistance [140]. More precisely, the upregulation of EMT-related molecules offsets various mechanisms that inevitably lead to enhanced metastasis, invasion, and chemoresistance. Nevertheless, despite considerable evidence that implicated Hh signaling in EMT regulation and carcinogenesis, its role in EMT-mediated chemoresistance has not been elucidated until much more recently.

Several preclinical studies have suggested the role of Hh in the EMT-mediated 5-FU resistance in CRCs. For instance, a recent study by Zhang et al. revealed GLI1 to be significantly overexpressed in 5-FU resistant CRC cells exhibiting EMT phenotype [80]. Notably, the 5-FU resistant CRC LoVo cell line showed a marked increase in invasiveness. *GLI1* knockdown significantly reduced the expression of mesenchymal (Vimentin and SNAI1) markers, invasiveness, and the sensitized 5-FU resistant CRC LoVo cell line to 5-FU treatment [80], suggesting EMT regulation to be the major function of GLI1 in mediating 5-FU resistance in CRC. Similarly, in a separate study by Liu et al., the 5-FU resistant colon HCT-8 cancer cell line showed increased EMT signatures such as reduced cell–cell adhesion and E-cadherin expression, increased vimentin expression, and enhanced migration. Consistent with findings by Zhang et al., the inhibition of Hh signaling at the SMO level with GDC0449 was found to reverse EMT signals and 5-FU resistance, as shown by N-cadherin downregulation and increased 5-FU sensitivity [141].

Besides 5-FU, the acquisition of EMT or mesenchymal phenotype has also been shown to promote cisplatin resistance in cancers. An initial experiment by Ahmad et al. revealed that the transcriptional upregulation of Shh by TGF-β1 induced EMT phenotype in the non-small cell lung carcinoma (NSCLC) A549 cell line [142], and further study by the researchers showed that the inhibition of Hh signaling with GDC0449 or siRNA-mediated *Shh* knockdown sensitized these EMT cells to cisplatin. Interestingly, parental cells that did not express appreciable levels of Shh were not sensitized to cisplatin upon the same treatment, suggesting a major role of Hh signaling in cisplatin resistance. Furthermore, it was revealed that the two major negative regulators of the EMT-regulating miRNA families, miR-200b and let-7c, were significantly downregulated in these Hh-overexpressing EMT cells. Of note, EMT transcription factors such as ZEB1 have been shown to negatively regulate mir-200b expression [143], while TWIST1 and SNAI1 suppressed let-7 expression [144,145]. Hh signaling inhibition with GDC0449 restored both the miRNAs’ expression, which was accompanied by increased epithelial (E-cadherin) and reduced mesenchymal (ZEB1) markers as well as reduced cisplatin resistance. Additionally, the researchers demonstrated that the downregulation of these miRNAs in GDC-0449 treated EMT cells abrogated the reduction in drug resistance induced by GDC-0449 [146], further enforcing the role of Hh-mediated chemoresistance via miRNA regulation.

As mentioned above, mesenchymal cells can acquire chemoresistance by the upregulation of transporters, and both EMT and transporters are known to be regulated by Hh signaling. In Huh-7 and HLE DN hepatoma subpopulations, higher Hh pathway (PTCH1, GLI1, GLI2) activity was associated with enhanced invasive capability, EMT acquisition (decrease E-cadherin and increase vimentin), and increased ABC transporter (ABCC1 and ABCB2) expression, resulting in enhanced chemoresistance [132,133,134]. In breast cancer cell lines, OPN induced mesenchymal phenotype by the upregulation and downregulation of several mesenchymal (N-cadherin, vimentin, TWIST, and SLUG) and epithelial (E-cadherin and keratin-18) markers, respectively, through noncanonical regulation of GLI1 (discussed in Section 3.5). Additionally, the acquisition of mesenchymal phenotype was associated with enhanced ABC transporters (ABCB1 and ABCG2), drug retention, and chemoresistance. Conversely, the silencing of GLI or OPN reversed EMT by reducing mesenchymal markers and increasing epithelial marker (keratin 18) expression, decreased ABCB1 and ABCG2 expression, and restored chemosensitivity to cancer chemotherapeutics [126]. Overall, these results suggest a dual role of GLI (canonical and noncanonical) in promoting chemoresistance by conferring EMT phenotype and regulating ABC transporters. Hh signaling promotes EMT and concomitantly upregulates transporters in mesenchymal cells to promote chemoresistance, while mesenchymal cells may also utilize EMT-related factors to upregulate various drug resistance mechanisms, including transporters [140] and drug-metabolizing enzymes (e.g., ALDH, cytochrome P450s) [147] to further enhance chemoresistance.

One of the mechanisms by which EMT or EMT-inducible transcription factors may promote chemoresistance is through the induction of tumor-initiating cells or CSCs [148]. Initial studies by Zhan et al. revealed that elevated GLI1 contributed to acquired resistance and mesenchymal phenotypes in doxorubicin-selected multidrug-resistant chronic myelogenous leukemia (CML) K562 and vincristine-selected multidrug-resistant human cervical epidermoid carcinoma (KB) sublines [149]. Further study by the same group revealed that GLI1 regulates the expression of EMT-inducing transcription factors, TWIST1 and SNAI1, to promote tumor-initiating cell-like properties and consequently chemoresistance in the resistant sublines. Evidently, TWIST1 and SNAI1 levels were markedly elevated in these multidrug-resistant sublines exhibiting heightened tumor-initiating potential. The inhibition of Hh signaling with cyclopamine or GANT-58 or the knockdown of *TWIST1* and *SNAI1* transcription factors attenuated the tumor-initiating cell properties of these multidrug-resistant sublines, as shown by reduced clonogenic, self-renewal, and sphere-formation ability, and restored their chemosensitivity to doxorubicin and vincristine. Mechanistically, ChiP and luciferase reporter analysis revealed that GLI1 transcriptionally regulates the expression of the EMT-inducing transcription factors, *TWIST1* and *SNAI1*, by binding to the putative GLI1 binding sequence CATCACCCA within their promoter regions to promote tumor-initiating-like properties and consequently chemoresistance. Additionally, constitutive activation of SMO in chemosensitive parental KB cells forcefully induced the expression of TWIST1 and SNAI1, which was associated with increased tumor-initiating cell properties as well as increased doxorubicin and vincristine resistance, and this effect can be reversed upon *TWIST1* and *SNAI1* knockdown [150]. In support of these findings, Mani et al. also reported that ectopic expression of either TWIST1 or SNAI1 bestowed EMT and tumor-initiating cell properties in human mammary epithelial cells [151]. Additionally, high expression of TWIST1 and SNAI1 has been shown to upregulate the expression of CSC markers such as ALDH, SOX2, and OCT4 [152,153]. Interestingly, TWIST1 and SNAI1 mediated chemoresistance was independent of ABC transporters; the knockdown of *TWIST1* and *SNAI1* had no effect on ABCB1 and ABCG2 expression [150], suggesting the gain of tumor-initiating capabilities to be the major contributor to acquired chemoresistance.

Similarly, Hh signaling was found to regulate EMT in PANC-1 tumorspheres exhibiting stem-like properties to promote metastasis and chemoresistance. Evidently, SMO was overexpressed in PANC-1 tumorspheres and was found to upregulate the expression of mesenchymal markers SNAI1 and N-cadherin while concomitantly downregulating E-cadherin. The loss of epithelial and gain of mesenchymal characteristics, a key feature of EMT, is an integral step needed for cancer cells to invade and metastasize. Notably, increased mesenchymal features of PANC-1 tumorspheres were associated with enhanced Transwell invasion in vitro and increased pulmonary metastasis in vivo in nude mice. Additionally, these tumorspheres displayed stronger resistance to gemcitabine. Conversely, when *SMO* was knocked down in the tumorspheres, they displayed a decreased capacity for invasion and pulmonary metastasis as well as increased sensitivity to gemcitabine [154].

**Figure 5:**
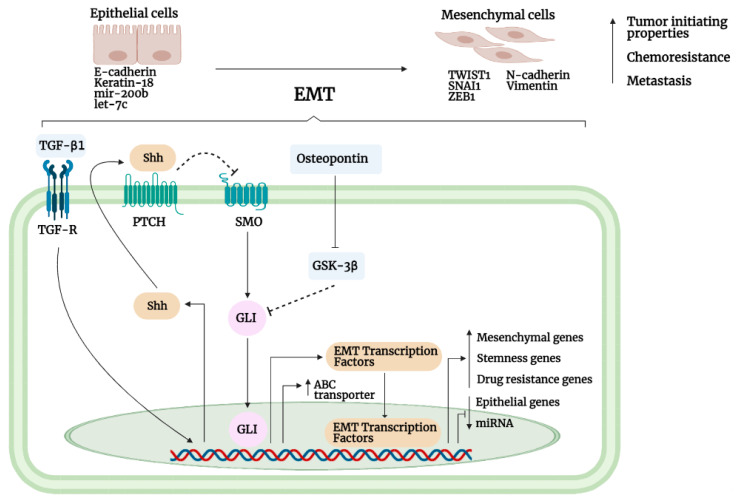
A schematic representation of canonical and noncanonical GLI regulation in promoting EMT and chemoresistance. TGF-β1 stimulation induced the transcriptional upregulation of Shh, which activates canonical Hh-GLI signaling in an autocrine manner. On the other hand, OPN inactivates GSK3β, promoting the activation of GLI independent of canonical input. Activated GLI translocates into the nucleus, where it upregulates the expression of EMT transcription factors (e.g., *TWIST1*, *SNAI1*, *ZEB1*). In turn, upregulated EMT transcription factors induce the expression of mesenchymal (e.g., *N-cadherin*, *Vimentin*), stemness (e.g., *SOX2*, *OCT4*, *ALDH*), and drug resistance genes (e.g., *ALDH*, ABC transporters) while concomitantly downregulating epithelial (*E*-*cadherin*) genes and miRNAs (mir-200b and let-7 family). Consequently, this results in the formation of mesenchymal cells with tumor-initiating-like properties and the increased capability to metastasize and resist cytotoxic chemotherapeutics. Additionally, increased GLI1 activity also upregulates ABC transporters, further enhancing chemoresistance by increasing drug efflux in mesenchymal cells.

Taken together, these results suggest a complex interplay between Hh signaling, EMT, transporter expression, and tumor-initiating properties in promoting chemoresistance. In the same cell, Hh signaling can promote EMT and upregulate transporters, while upregulated EMT-transcription factors can also bestow tumor-initiating or CSC-like traits or enhanced expression of drug-resistance proteins in the cell, which together may greatly enhance tumorigenesis and resistance to chemotherapeutics. Figure 5 summarizes the described mechanisms which canonical Hh-GLI signaling or non-canonically activated GLI utilizes to promote EMT and consequently chemoresistance discussed in this section.

### 3.7. Cancer Stem Cells

CSCs represent a small subpopulation of cells within tumors that possess self-renewal, differentiation, tumorigenicity, and chemoresistance capabilities [155]. Although the existence of CSCs was highly controversial in the past, recent technological advancement has allowed the identification of novel CSC cell surface markers such as CD44, CD24, and CD133 for the isolation and enrichment of CSCs from tumor bulks of different tissue origins [156], which opens up new avenues in cancer and stem cell research. To date, CSC research’s major focus still includes finding potential molecular targets for the eradication of CSCs and uncovering underlying mechanisms by which CSCs resist chemotherapeutics and repopulate cancer cells. However, the cellular mechanisms regulating CSC maintenance still remain unclear, although accumulating evidence has implicated key developmental signaling pathways such as Hh that have shown a promising role in stem cell regulation [58]. Due to its major role in regulating the self-renewal capacity of embryonic, adult, and progenitor stem cells, Hh has been heavily implicated in CSC maintenance, despite it being a newly established pathway in development and oncogenesis. More importantly, the CSC population’s maintenance as a result of Hh pathway overexpression has resulted in therapy failure and tumor repopulation, which renders chemotherapy ineffective in the complete eradication of cancer cells. This is because as cells acquired CSC characteristics, they acquired enhanced capacity for DNA repair, decreased cell cycle arrest and apoptosis, and ABC transporter expression, which results in enhanced resistance to chemotherapeutics. Additionally, their relatively quiescent state makes them a difficult target for conventional therapeutics aimed at killing fast-growing cells.

A recent study by Yoon et al. demonstrated that the Hh pathway plays a critical role in maintaining CD44+ gastric CSC subpopulation, which in turn leads to enhanced chemoresistance [157]. CD44, a novel CSC marker, has been used to identify and isolate CSC subpopulations. GC cell lines AGS, MKN-45, and N87 grown as spheroids were found to overexpress CD44 and GLI1, which was associated with the increased expression of self-renewal proteins SOX2 and NANOG. Conversely, pharmacological inhibition of Hh signaling with vismodegib reduced CSC traits, as shown by the marked reduction in the spheroid formation and CD44+ expression. CSCs are known to possess EMT characteristics (discussed in Section 3.6), and the inhibition of Hh signaling was also shown to decrease migration, invasion, and anchorage-independent growth capability. Notably, the CD44+ GC cell lines isolated from spheroids were significantly more resistant to the cytotoxicity effect of 5-FU and cisplatin than CD44- cell lines, and treatment with vismodegib or SMO shRNA sensitizes CD44+ but not CD44- cells to 5-FU and cisplatin in both in vitro cell lines and in vivo mice xenografts. Furthermore, in a randomized phase II study, several GC patients that showed high CD44 median score had a complete response when treated concurrently with vismodegib, 5-FU, oxaliplatin, and leucovorin, but the combination treatment was not effective in patients expressing low CD44 median score, suggesting a major role of Hh signaling in CSC-mediated chemoresistance [157].

Similarly, in a different study by Song et al., tumorsphere cells derived from GC cell lines HGC-27, MGC-803, and MKN-45 were also characterized by significantly higher Hh pathway activity (Shh, PTCH1, SMO, and GLI1) and CD44+ expression than adherent cells [158]. The inhibition of Hh signaling in the CD44+ tumorsphere cells resulted in a marked reduction in self-renewing capacity, as shown by the decreased sphere- and colony-forming capacity. Moreover, the tumorsphere cells derived from GC cell lines and primary tumor samples exhibited enhanced resistance towards chemotherapeutics oxaliplatin and mitomycin. Combination treatment of the SMO inhibitor 5E1 with the chemotherapeutics resulted in an enhanced overall cell death rate compared to treatment with any of the agents alone [158]. Besides CD44+, Yu et al. also reported that tumorspheres derived from N87 cells demonstrated elevated levels of CSC markers CD90 and CD133, increased sphere-forming efficiency, and enhanced resistance towards cisplatin, all of which could be reduced upon *GLI1* knockdown [128].

The Hh pathway was also reported to be involved in the regulation of esophageal CSC traits to promote chemoresistance. Initial experiments by Wang et al. revealed Hh members Shh and PTCH1 to be enriched in the EC resection material of patients with micro residual disease after receiving neoadjuvant chemoradiation, suggesting a role of the Hh pathway in mediating chemoradiotherapy resistance [159]. Further investigation using in vitro CD44+ OE21 and OE33 cell lines sorted using fluorescence-activating cell sorting (FACS) showed high expression of *PTCH1* based on qPCR analyses compared to their differentiated counterparts, which was associated with increased CSC traits such as enhanced sphere-forming ability and carboplatin and photon irradiation resistance. Indeed, the inhibition of the Hh pathway with vismodegib decreased CD44+ CSC phenotype and sphere-forming potential in the EC cells, thus improving therapeutic response to carboplatin and radiation [159].

Similarly, a study by Cui et al. also showed that higher Shh protein expression was strongly associated with a poorer outcome in ESCC patients who underwent platinum-based regimens [125]. Moreover, continuous treatment with cisplatin enhanced Hh signaling in ESCC cell lines TE-1 and KYSE510, which was associated with the upregulation of CSC-related transcription factors (NANOG, SOX2, and OCT4) and enhanced tumorsphere formation. Notably, the treatment of DHA suppresses the activation of the Hh pathway that led to a decrease in both cell proliferation and CSC traits, thus sensitizing ESCC to the cytotoxicity effect of cisplatin in both in vitro ALDH+ TE-1 cells and in vivo BALB/c nude mice bearing TE-1 xenograft models. As mentioned previously, CSCs are known to employ transporters to promote the efflux of drugs, including chemotherapeutics. Upregulated Shh signaling was also shown to increase the expression of ABCB1, which in turn reduced the cellular enrichment of cisplatin and promoted chemoresistance [125]. These results suggest that Hh signaling can induce CSC characteristics while promoting chemoresistance, in part, through the upregulation of ABC transporters.

Hh signaling is well-known to play a pivotal role in regulating stem cell and progenitor cell expansion in hematopoiesis. In particular, an earlier study by Kobune et al. demonstrated that Indian hedgehog (Ihh) but not Shh was overexpressed in cord blood (CB) CD34+ hematopoietic progenitor cells (HPCs) [160]. Furthermore, Ihh secreted from primary stromal cells was also found to regulate the proliferation of CD34+ hematopoietic progenitor cells and short-term myeloid- and lymphoid-repopulating cells [160]. In light of these findings, a later study by the researchers revealed that mostly Ihh and its downstream effectors (GLI1 and GLI2) were active in several human acute myeloid leukemia (AML) cells, including primary CD34+ leukemic cells and cytokine-responsive CD34+ Kasumi-1, Kasumi-3, and TF-1 cell lines. More importantly, these CD34+ cells were resistant to Ara-C treatment, a conventional chemotherapy drug used to treat acute leukemia. Subsequently, the inhibition of the Ihh autocrine loop with 5E1 or cyclopamine induced apoptosis and sensitized the CD34+ cells to Ara-C [161]. Similarly, a recent study by Long et al. revealed that GLI1 was primarily enriched in CD34+ enriched AML Kasumi-1 and KG1a progenitor cells, and the inhibition of GLI1 with GANT61 reduced colony-forming capacity and induced apoptosis in these progenitor cells [162]. Moreover, GANT-61 treatment sensitized CD34+ primary AML cells to Ara-C, as shown by a significantly higher reduction in cell survival compared to CD34- AML cells [162].

Studies have also implicated the role of Hh in the regulation of pancreatic CSCs and, consequently, gemcitabine resistance. An earlier study by Yao et al. revealed that the expression levels of Hh members (Shh, SMO, and GLI1) and CSC markers (CD44 and CD133) were highly expressed in gemcitabine-resistant PC cell lines SW1990 and CFPAC-1, which can be downregulated upon cyclopamine treatment [163]. In light of this, a later study by Huang et al. demonstrated that tumorspheres derived from the PANC-1 cell line possess high self-renewal, differentiation, and tumorigenic potential, as well as increased CSC marker CD133 and Hh pathway (SMO, GLI1, and GLI2) activity compared to their parental cells. PANC-1 tumorspheres were innately resistant to the cytotoxic effect of 5-FU and gemcitabine, and treatment of the tumorspheres with cyclopamine inhibited their proliferative and self-renewal potential as well as sensitized them to the cytotoxicity effect of chemotherapeutics [131].

Interestingly, a novel molecular link between Hh and TUSC3 had been proposed by Ren et al., whereby Hh signaling serves as a mediator of TUSC3-induced CRC stemness to promote drug resistance [164]. A previous study by the same group revealed that TUSC3 was overexpressed in human CRC tissues and played an oncogenic role in CRC progression in vitro and in vivo [165]. Further study revealed that elevated TUSC3 expression was associated with shorter OS and disease-free survival (DFS) in CRC patients. Additionally, TUSC3-overexpressed RKO and CACO2 CRC cell lines were associated with increased CSC traits such as increased CD133 and sphere-forming efficiency, all of which enhanced the drug resistance of cells to both 5-FU and cisplatin. Further analyses revealed the Hh pathway to be a mediator of TUSC3-induced CSC phenotype and drug resistance in the CRC cell lines. Evidently, the inhibition of the Hh pathway with GANT-61 downregulated the expression of SMO and GLI1 and reversed CSC traits, which led to reduced 5-FU and cisplatin drug resistance. To further support the role of Hh signaling as a mediator of TUSC-induced drug resistance, co-immunoprecipitation (Co-IP) and immunofluorescence (IF) study demonstrated a tight interaction and spatial overlap between SMO and TUSC3 protein, but not between TUSC3 and CD133 [164]. In support of this finding, numerous studies have also established Hh activation as an inducer of CD133 expression in CSCs [58], including colorectal CSCs [166]. This would suggest that the TUSC3 role has SMO as an intermediate in the pathway to promote CSC phenotype, leading to chemoresistance. Additionally, TUSC3 overexpression was also found to upregulate the expression of ABCC1, presumably through Hh pathway activation, suggesting that Hh signaling may promote chemoresistance in CRC stem-like cells, in part, through ABCC1 upregulation [164].

Interestingly, it was found that the hypoxic TME condition contributed to the chemoresistance of colorectal cancer stem cells. HIF-1α production induced by hypoxic conditions was shown to cooperate with CAFs to increase the expression of TGF-β2, which non-canonically activated the transcription of *GLI2* in cancer stem cells, thus leading to increase stemness, dedifferentiation, and resistance to FOLFOX (combination of 5-FU and oxaliplatin) chemotherapy. Additionally, the HIF1a-TGF-β2-GLI2 axis was associated with higher tumor recurrence following chemotherapy treatment. Indeed, the co-treatment of TGF-β inhibitor SD208 and GLI inhibitor GANT61 effectively reversed 5-FU and oxaliplatin chemoresistance in tumorsphere cells grown in CAF-conditioned medium and CT128 patient-derived xenograft, as well as significantly reduced the expression of CSC genes and stemness markers (*POU5F1*, *SOX2*, *ALDH1A1*, and *ALDH1A3*) [167].

### 3.8. Section Summary

Hh signaling plays an important role in the oncogenesis of various cancers. In addition to its tumor-promoting role, numerous studies have also implicated a significant role of Hh signaling in promoting the chemoresistance of cancers via the regulation of various intrinsic drug resistance mechanisms (as discussed earlier; Table 1). More importantly, the synergistic use of Hh inhibitors and conventional chemotherapeutic drugs has proven fruitful in overcoming the chemoresistance of multiple cancers in preclinical studies and a handful of clinical studies (Table 1). Thus, targeting Hh signaling may be a suitable approach for managing Hh-overexpressing cancers in the settings of chemotherapy to improve clinical response and, if possible, mitigate chemoresistance.

## 4. Conclusions

Overall, our review has provided a substantial role of Hh signaling in promoting cancer chemoresistance through the regulation of multiple mechanisms. Firstly, evidence has supported the role of Hh signaling in the regulation of DNA-repair enzymes at the transcriptional level for the reparation of chemotherapeutics-induced genotoxicity, especially in the context of glioma. Secondly, firm evidence has implicated the role of Hh signaling in the regulation of transporter-mediated drug efflux to promote chemoresistance, especially since many transporters are direct transcriptional targets of GLIs. Thirdly, Hh signaling induces EMT in cells by the regulation of EMT transcription factors, which promotes mesenchymal and tumor-initiating-like properties as well as upregulates transporters, all of which contribute to enhanced chemoresistance. Fourthly, it is evident that Hh signaling plays an important role in forming CSC niches that are innately more resistant to chemotherapeutics. Lastly, recent evidence has suggested the role of Hh signaling in the regulation of autophagy, drug inactivation, and TME; however, more studies are still required to confirm their relevance in the context of chemoresistance.

Based on the evidence from multiple lines of studies, we propose that targeting Hh-GLI signaling, especially GLI1, holds promise for the mitigation of chemoresistance. Combining both Hh inhibitors and conventional cancer therapeutics in cancer therapy is proven to be useful for the chemosensitization of tumors in both in vitro and in vivo studies. However, there is still a lack of clinical data to support the appropriate use of Hh inhibitors in the clinical settings of chemotherapy, maybe in part due to the undesired toxicity effect of currently available Hh inhibitors. Additionally, an important aspect that has not been explored is the non-canonical regulation of GLI in the context of chemoresistance, and the non-canonical route has been proven to be vital for the survival of many GLI-dependent cancers. Despite the abundance of evidence that suggests the Hh pathway as a potential target in overcoming cancer growth, many areas concerning Hh signaling regulation and its role in regulating drug resistance mechanisms to promote chemoresistance are still relatively understudied. Thus, a further and more careful elucidation of these areas will be required before we can solidify their relevant use in the clinical settings of chemotherapy.

## Figures and Tables

**Table 1:** Summary of the described studies on the Hh regulation of drug resistance mechanisms and the use of combination drug therapy involving Hh inhibitors and chemotherapeutic agents and its treatment outcome.

Molecular Target	Drug	Status	Cancer Target	Drug Combination	Mechanism of Chemoresistance	Treatment Outcome	Citation
Shh/Ihh	5E1	Preclinical study	Gastric	Oxaliplatin and Mitomycin	Hh signaling enhanced self-renewing capacity of tumorspheres, which promotes oxaliplatin and mitomycin resistance	Hh signaling inhibition enhanced cell death of CD44+ HGC-7 tumorsphere cells and primary gastric tumor samples when co-treated with oxaliplatin and mitomycin	[158]
SMO	Sonidegib/Erismodegib (NVP-LDE-225)	Preclinical study and phase I clinical trail EDALINE	Breast	Docetaxel	M6-Hh cells activate CAFs via paracrine Hh signaling to induce ECM remodeling and consequently chemoresistant CSCs phenotype in adjacent epithelial cells	SMO inhibition reduced collagen fibrillar deposition and consequently reversed the CSC phenotype of M6-Hh cells, which sensitizes M6-Hh tumors, patient-derived xenografts, and metastatic TNBC patients to docetaxel	[105,106]
		Preclinical study	Ovarian	Paclitaxel	The upregulation of SMO, GLI1, and GLI2 modulate ABCB1 expression to promote paclitaxel resistance	SMO inhibition sensitized taxane resistant ALDH-negative and positive A2780 and SKOV3 cell lines to paclitaxel by downregulating ABCB1 expression	[122]
	Vismodegib (GDC-0449)	Preclinical study	Myeloid Leukemia	Ribavirin, cytarabine, methotrexate, venetoclax, 5-fluorouracil, sunitinib, and idarubicin	High levels of GLI1 mediated the UGT1A-dependent glucuronidation of several cancer therapeutics via calreticulin-dependent UGT1A protein stabilization to enhance drug elimination	Inhibition of GLI1 reduced UGT1A dependent glucuronidation of cancer therapeutics to promote drug accumulation (e.g., eIF4E-ribavirin complex) in primary AML specimens and acquired resistant cell lines	[114,117]
			Colon	5-fluorouracil	Elevated GLI1 levels induced EMT phenotype, which was associated with 5-fluorouracil resistance	Inhibition of SMO reversed EMT signals and 5-fluorouracil resistance in colon cancer HCT-8 cell line	[141]
			Lung	Cisplatin	Elevated Hh signaling contributed to TGF-β1 induced EMT and cisplatin resistance via the downregulation of miR-200b and let-7c	The inhibition of Hh signaling sensitized A549 EMT cells to cisplatin through the restoration of miR-200b and let-7c expression and reversal of EMT genes	[146]
			Esophageal	Carboplatin	High levels of Shh and PTCH1 were associated with increased CSC traits and therapeutic resistance	Inhibition of Hh pathway via SMO decreased CD44+ CSC phenotype and sphere-forming potential in esophageal cancer OE21 cell line, which restored carboplatin sensitivity	[159]
		Preclinical study and phase II clinical trial	Gastric	5-fluorouracil, cisplatin, oxaliplatin, and leucovorin	Increased Hh signaling enhanced CSC traits in gastric cancer (GC) cells to promote 5-fluorouracil and cisplatin resistance	SMO inhibition sensitized CD44+ AGS, MKN45, and N87 spheroid cells and mice xenografts as well as GC patients with high CD44 median scores to the combination treatment of 5-fluorouracil and cisplatin and 5-fluorouracil, oxaliplatin, and leucovorin, respectively	[157]
	Saridegib (IPI-926)	Preclinical study	Pancreatic	Gemcitabine	Hh pathway activation enhanced stromal desmoplasia and hypovascularity to reduced gemcitabine delivery	SMO inhibition depletes stromal desmoplasia and increases MVD to enhance gemcitabine delivery and consequently intracellular dFdCTP accumulation in KPC mice tissues	[111]
	Cyclopamine	Preclinical study	Pancreatic	Gemcitabine and 5-fluorouracil	Hh signaling induced gemcitabine resistance through CSC induction, as well as ABCB1 and ABCG2-mediated drug efflux	Inhibition of SMO restored gemcitabine sensitivity in acquired gemcitabine-resistant SW1990 and CFPAC cell lines expressing CD44 and CD133, as well as restored gemcitabine and 5-fluorouracil sensitivity in PANC-1 tumorspheres by inhibiting their self-renewing capacity and ABCB1 and ABCG2 expression	[131,163]
			Glioma	Temozolomide	GLI1 upregulates *MGMT* at the promoter level to promote TMZ resistance	SMO inhibition restricts Hh/GLI signaling to downregulate *MGMT* expression and consequently improved TMZ toxicity in GBM U251 and U87 cell lines and xenografts	[75]
			Myeloid Leukemia	Cytarabine	Ihh and its downstream effector (GLI1 and GLI2) were enriched in CD34+ subpopulations, which was associated with cytarabine resistance	Inhibition of Ihh autocrine Hh signaling via SMO induced apoptosis and sensitized cytokine responsive CD34+ Kasumi-1, Kasumi-3, and TF-1 cell lines to cytarabine treatment	[161]
GLI1/2	GANT-58	Preclinical study	Myeloid leukemia and cervical	Doxorubicin and vincristine	GLI1 transcriptionally upregulates *TWIST1* and *SNAI1* expression to promote tumor-initiating properties and consequently chemoresistance	GLI1 inhibition reduced TWIST1 and SNAI1 levels, which restored the chemosensitivity of multidrug-resistant chronic myelogenous leukemia K562 and human cervical epidermoid carcinoma KB sublines to doxorubicin and vincristine	[150]
	GANT-61	Preclinical study	Myeloid Leukemia	Vincristine	High GLI1 levels upregulate ABCB1 to promote vincristine resistance	GLI1 inhibition reversed vincristine resistance of Lucena-1 cell line by interfering with ABCB1 expression	[32]
				Cytarabine	GLI1 enrichment in CD34+ cells enhanced colony-forming capacity and cytarabine resistance	GLI1 inhibition induced a significant reduction in cell viability of CD34+ compared to CD34- primary AML cells when treated with cytarabine	[162]
			Ovarian	Cisplatin	GLI2 transcriptionally upregulates *ABCB1* to promotes cisplatin resistance	GLI2 inhibition reduced ABCB1 levels, which enhanced cisplatin-induced DNA damage in cisplatin-resistant OC SK-OV-3 cell line	[123]
			Glioma	Temozolomide	High levels of GLI and MGMT expression in glioblastoma cells lead to TMZ resistance	Inhibition of GLI with GANT-61 reduced the level of MGMT, which restored the sensitivity to temozolomide in GBM U251 and U87 cell lines	[73]
			Hepatoma	Cisplatin, doxorubicin, and sorafenib	The binding of GLI1/2 to the GLI1-binding consensus sequence within the *ABCB2* promoter initiated its transcription to confer drug resistance	Inhibition of GLI1/2 downregulated the ABCB2 expression in Huh-7 DN (CD133-/EpCAM-) and trans (CD133−/EpCAM− transwell-selected) hepatoma subpopulations, which enhanced their chemotherapeutic drug sensitivity	[132]
			Colorectal	5-fluorouracil and cisplatin	Hh signaling has SMO as a mediator of TUSC3-induced CSC phenotype and drug resistance	Inhibition of Hh signaling at the GLI level inhibited the expression of CD133 and ABCC1 and decreased the number of TUSC3-overexpressing CACO2 and RKO2 tumorspheres, which was associated with increased 5-fluorouracil and cisplatin sensitivity	[164]
	SIR-38832	Preclinical study	Colorectal	5-fluorouracil	GLI1 transcriptionally upregulates *NBS1* and consequently MRN complex function to reduce 5-fluorouracil induced DNA damage	Inhibition of GLI1 activity reduced total NBS1 levels and impaired MRN complex function to increase 5-fluorouracil induced DNA damage in HT29 cell lines and xenografts	[81]
Not specified	Dihydroartemisinin	Preclinical study	Esophageal	Cisplatin	Hh activation upregulates ABCB1 levels to reduce cisplatin enrichment	Hh inhibition reduced ABCB1 levels and consequently enhanced cisplatin accumulation in the TE-1 cell line	[125]
HIF-1α	Oroxylin A	Preclinical study	Glioma	TMZ	HIF-1α activates Shh/GLI1/MGMT signaling to promote TMZ resistance	HIF-1α degradation inhibits Hh pathway activation and increases SUFU expression, thus reducing MGMT levels and restoring TMZ sensitivity in glioma U251 and C6 cell lines and xenografts	[75]
